# Association Between Hydrological Conditions and Dengue Fever Incidence in Coastal Southeastern China From 2013 to 2019

**DOI:** 10.1001/jamanetworkopen.2022.49440

**Published:** 2023-01-04

**Authors:** Chuanxi Li, Zhendong Wang, Yu Yan, Yinan Qu, Liangyu Hou, Yijie Li, Cordia Chu, Alistair Woodward, Tamara Schikowski, Paulo Hilário Nascimento Saldiva, Qiyong Liu, Qi Zhao, Wei Ma

**Affiliations:** 1 Department of Epidemiology, School of Public Health, Cheeloo College of Medicine, Shandong University, Jinan, China; 2 Shandong University Climate Change and Health Center, Shandong University, Jinan, China; 3 Dezhou Center for Disease Control and Prevention, Dezhou, China; 4 Centre for Environment and Population Health, School of Medicine, Griffith University, Nathan, Queensland, Australia; 5 Department of Epidemiology and Biostatistics, School of Population Health, Faculty of Medical and Health Sciences, University of Auckland, Auckland, New Zealand; 6 Department of Epidemiology, IUF-Leibniz Research Institute for Environmental Medicine, Düsseldorf, Germany; 7 Faculty of Medicine, University of São Paulo, São Paulo, Brazil; 8 State Key Laboratory of Infectious Disease Prevention and Control, National Institute for Communicable Disease Control and Prevention, Chinese Center for Disease Control and Prevention, Beijing, China; 9 Department of Vector Control, School of Public Health, Cheeloo College of Medicine, Shandong University, Jinan, China

## Abstract

**Importance:**

Dengue fever is a climate-sensitive infectious disease. However, its association with local hydrological conditions and the role of city development remain unclear.

**Objective:**

To quantify the association between hydrological conditions and dengue fever incidence in China and to explore the modification role of city development in this association.

**Design, Setting, and Participants:**

This cross-sectional study collected data between January 1, 2013, and December 31, 2019, from 54 cities in 4 coastal provinces in southeast China. The Standardized Precipitation Evapotranspiration Index (SPEI) was calculated from ambient temperature and precipitation, with SPEI thresholds of 2 for extreme wet conditions and −2 for extreme dry conditions. The SPEI–dengue fever incidence association was examined over a 6-month lag, and the modification roles of 5 city development dimensions were assessed. Data were analyzed in May 2022.

**Exposures:**

City-level monthly temperature, precipitation, SPEI, and annual city development indicators from 2013 to 2019.

**Main Outcomes and Measures:**

The primary outcome was city-level monthly dengue fever incidence. Spatiotemporal bayesian hierarchal models were used to examine the SPEI–dengue fever incidence association over a 6-month lag period. An interaction term between SPEI and each city development indicator was added into the model to assess the modification role of city development.

**Results:**

Included in the analysis were 70 006 dengue fever cases reported in 54 cities in 4 provinces in China from 2013 to 2019. Overall, a U-shaped cumulative curve was observed, with wet and dry conditions both associated with increased dengue fever risk. The relative risk [RR] peaked at a 1-month lag for extreme wet conditions (1.27; 95% credible interval [CrI], 1.05-1.53) and at a 6-month lag for extreme dry conditions (1.63; 95% CrI, 1.29-2.05). The RRs of extreme wet and dry conditions were greater in areas with limited economic development, health care resources, and income per capita. Extreme dry conditions were higher and prolonged in areas with more green space per capita (RR, 1.84; 95% CrI, 1.37-2.46). Highly urbanized areas had a higher risk of dengue fever after extreme wet conditions (RR, 1.80; 95% CrI, 1.26-2.56), while less urbanized areas had the highest risk of dengue fever in extreme dry conditions (RR, 1.70; 95% CrI, 1.11-2.60).

**Conclusions and Relevance:**

Results of this study showed that extreme hydrological conditions were associated with increased dengue fever incidence within a 6-month lag period, with different dimensions of city development playing various modification roles in this association. These findings may help in developing climate change adaptation strategies and public health interventions against dengue fever.

## Key Points

**Question:**

Are local hydrological conditions associated with dengue fever incidence, and do city development indicators modify this association?

**Findings:**

In this cross-sectional study of 54 cities in China, extreme hydrological conditions were associated with an increased risk of dengue fever, and different characteristics of city development played a role in modifying the association between hydrological conditions and dengue fever incidence in various ways.

**Meaning:**

The findings of this study suggest that hydrological conditions are associated with dengue fever incidence, and the findings may inform climate change adaptation strategies and public health interventions against dengue fever.

## Teaser

This cross-sectional study examines the risks and timing of dengue virus transmission in regions with extreme weather patterns and areas with varying levels of economic development.

## Introduction

Dengue fever is a mosquito-borne disease mostly affecting the tropical and subtropical regions of the world. There are 100 to 400 million infections per year,^1^ making dengue fever one of the top 10 health threats worldwide.^2^ China has had substantial achievements in controlling dengue fever in the past decades, with a major epidemic confined to its low- and middle-latitude coastal areas. However, incidence of dengue fever has rebounded in recent years, and the high-risk areas have expanded to higher latitudes,^3^ causing considerable public health burden. In 2014, over 45 000 infections were reported in Guangdong province in southern China.^4^ In 2017, an epidemic with over 200 infections was recorded in Shandong province in northern China.^5^ In the context of increasing dengue fever burden, clarifying its major risk factors and potential modifiers is important for population health promotion.

Climate change is a critical worldwide challenge that may alter temperature and precipitation patterns, increase the risk of floods and droughts, and, in the long-term, change the local hydrological conditions.^6,7^ Evidence has demonstrated that extreme weather events, such as floods, tropical cyclones, and droughts, play important roles in dengue fever incidence.^8,9^ Theoretically, seasonal or interannual change of hydrological conditions may also affect dengue fever incidence by altering the development of mosquitoes and vector-host contact. For example, the anomaly of wet conditions and associated precipitation may enrich the static water source, which provides the ideal breeding habitat for *Aedes aegypti* (Linnaeus) mosquitoes, the primary vector of dengue virus transmission.^10^ Dry conditions, especially prolonged severe drought, might lead to increased use of semipermanent water storage containers, which are known to be critical larval habitats.^11^ Additionally, the precipitation surplus and deficit have been associated with different timing of dengue fever occurrence.^12,13^ Extreme precipitation may be associated with increased risk of dengue virus transmission within a few weeks, while drought may be associated with delayed risk for up to several months due to the gradual change in human coping behavior.^12,13^

To date, the majority of dengue fever cases occur in urban or semiurban areas, suggesting that city development may alter the magnitude of dengue fever incidence. Specifically, many factors may explain the difference in dengue fever incidence between urban and rural areas, such as mosquito abundance, population density, and dengue fever prevention practices.^14,15^ A Brazilian study^12^ found that, in highly urbanized areas with water shortages, the dengue fever risk was even higher during extreme dry conditions, while extreme wet conditions were associated with a higher risk of dengue fever in less urbanized areas. However, city development is a large concept in terms of public health, and it is inadequate to measure the implications for dengue fever using a single indicator. There are various dimensions that may modify the association between hydrological conditions (ie, the combination of local precipitation, ambient temperature, and evaporation) and dengue fever incidence following different pathways.^16,17^ For example, abundant health care resources may improve the emergency response capacity for dengue fever treatment,^18^ while high population density and mobility may increase the frequency of the mosquito-person transmission chain.^19,20^ In past decades, China experienced one of the world’s fastest urbanization and infrastructure development.^21,22^ However, limited research is available exploring the modification role of various dimensions of city development in the association between hydrological conditions and dengue fever incidence. Given the increasing dengue fever incidence and the extended geographic distribution in the context of climate change, it is necessary to explore the modification of the association between hydrological conditions and dengue fever incidence by multidimensional city features.

This cross-sectional study was performed to quantify the association between hydrological conditions and dengue fever incidence in 4 coastal southeastern provinces of China from 2013 to 2019 and to explore the modification role of city development in this association. The findings may provide evidence to support developing tailored prevention strategies to reduce the potential dengue fever risk under various hydrological conditions.

## Methods

Disease surveillance data were obtained from the National Notifiable Diseases Surveillance System without identifiable information. Exposure data were collected from publicly accessible data sets. Thus, this study was deemed exempt from ethical approval, and the informed consent requirement was waived by the Shandong University Institutional Review Board. We followed the Strengthening the Reporting of Observational Studies in Epidemiology (STROBE) reporting guideline.

### Study Area

**Figure 1.**
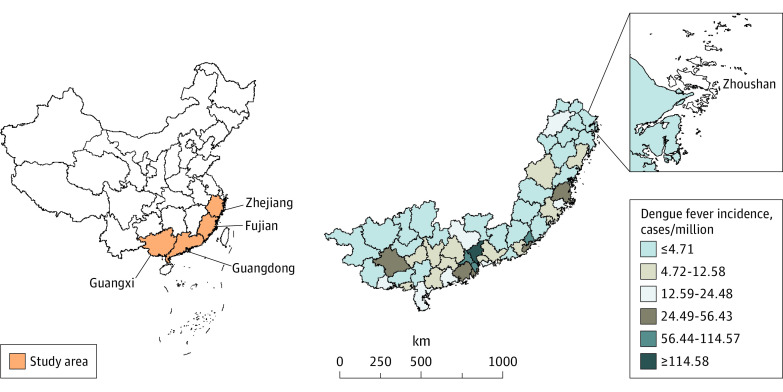
Geographic Locations and Spatial Distribution of Dengue Fever Incidence for the 54 Cities of the Four Provinces in China From 2013 to 2019

This population-based cross-sectional study included 54 of 55 cities in 4 coastal provinces in southeast China (Zhejiang, Fujian, Guangdong, and Guangxi) (Figure 1), where dengue fever cases accounted for 75% of national records between January 1, 2013, and December 31, 2019. The study excluded Zhoushan due to the lack of meteorological measurements for that city. The whole study area covers 0.65 million km^2^ and has 263 million residents. Due to a monsoon climate, this region is hot and rainy in the summer, warm and dry in the winter, and subject to hydrological extremes.

### Dengue Fever Cases

Dengue fever is classified as a notifiable B-category infectious disease in China, where all cases should be reported to local health care authorities within 24 hours of diagnosis.^23^ We identified dengue fever cases between January 1, 2013, and December 31, 2019, from the National Notifiable Diseases Surveillance System. All cases were diagnosed by clinical diagnosis and laboratory confirmation by professional medical institutions.^24,25^ We then calculated the city-level monthly dengue fever incidence as the main outcome.

### Exposure Assessment

For the accuracy and robustness of hydrological indices, we collected the monthly mean, maximum, and minimum temperatures and precipitation during an extended period (1961-2019) from the National Climate Center of China Meteorological Administration at a spatial resolution of 0.25° × 0.25°.^26^ This data set was developed via interpolating data from over 2400 weather stations, with its high-quality performance validated.^27,28^ The monthly meteorological data for each city was aggregated by computing the mean (temperature) or sum (precipitation) of the grids within the city boundary. The Standardized Precipitation Evapotranspiration Index (SPEI) was selected to measure the local hydrological conditions (eMethods in Supplement 1). Values greater than 0 indicate water surplus (wet), and values less than 0 represent water deficit (dry). Following the official standard in China and previous literature,^29,30^ we chose the SPEI threshold of 2 for extreme wet conditions and −2 for extreme dry conditions. The multiscalar character of SPEI enables it to depict the different types and outcomes of wet and dry conditions. We calculated the 3-month (SPEI-3), 6-month (SPEI-6), and 12-month (SPEI-12) SPEI on a monthly basis, which represented the seasonal, medium-term, and interannual hydrological conditions, respectively.^31,32^

**Figure 2.**
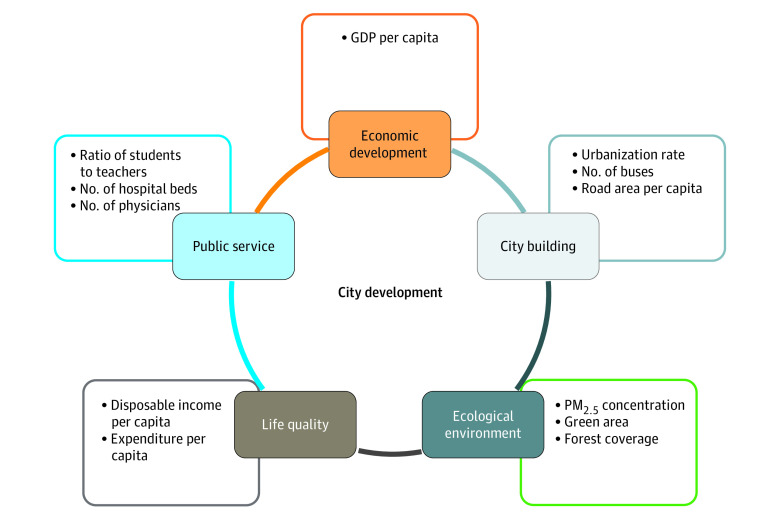
Framework for City Development Quality Evaluation The 12 indicators initially selected are listed. The full list of the 50 indicators is provided in eTable 1 in Supplement 1. GDP indicates gross domestic product; PM, particulate matter.

The Chinese government recommends the use of 50 indicators across 5 dimensions to evaluate city development (eTable 1 in Supplement 1).^33^ Considering the biological pathways to dengue virus transmission and data availability, we initially chose 12 metrics (Figure 2; eTable 2 and eFigure 1 in Supplement 1). We collected annual data on indicators for each city during the 2013 to 2019 study period using the *China City Statistical Yearbook*^34^ and the Tracking Air Pollution in China data set. Correlation analysis was performed to exclude highly correlated indicators (eTable 3 in Supplement 1). Ultimately, we selected 5 indicators covering all 5 dimensions, including gross domestic product (GDP) per capita, number of physicians, disposable income per capita, green area, and urbanization rate, and explored their potential modification roles in the association.

### Statistical Analysis

We constructed a spatiotemporal bayesian hierarchical mixed model combined with a distributed lag nonlinear model to quantify the association between SPEI and dengue fever incidence for up to 6 months, while controlling for structured and unstructured random effects.^12^ A negative binomial distribution was applied to deal with overdispersion. In the cross-basis function between SPEI and dengue fever incidence, natural cubic splines with 2 equally spaced internal knots were used for the exposure and lag (in the log scale) dimensions, respectively. The SPEI–dengue fever incidence association was described using relative risk (RR) and corresponding 95% credible interval (CrI) by comparing the risk at normal conditions (SPEI of 0). We calculated the lag-specific RR and cumulative RR over a lag of 6 months. The model parameters were estimated using integrated nested Laplace approximations.^35,36^

We used a stepwise strategy for model selection (eFigure 2 in Supplement 1). First, we fitted a baseline model comprising only provincial-specific monthly random effects and year-specific spatially random effects at the city level. Second, SPEI at different time scales (SPEI-3, -6, and -12) was introduced into the baseline model in turn. The goodness of fit of each model was evaluated using the deviance information criterion and the mean cross-validated log score, and the model with the lowest values was chosen. Third, other meteorological variables were entered into the chosen model to check their potential confounding. Fourth, a model with SPEI-3 and monthly minimum temperature was confirmed as best fitting and was applied for the formal analysis (eTable 4 in Supplement 1). To assess the modification role of city development in the SPEI–dengue fever incidence association, a linear interaction term between SPEI-3 and each development indicator was additionally entered into the final model. As with previous studies,^37,38^ in this study, the city development indicator in the interaction term was centered on its 10th, 50th, and 90th percentile of the 54 cities’ value range to extract the effect size estimates of SPEI-3 for dengue fever incidence at different development levels.

Sensitivity analyses were performed by changing the degrees of freedom of the SPEI-3 and meteorological variables as well as the position of knots for exposure or lag dimensions in the cross-basis function. Suboptimal combinations of hydrological and meteorological factors were also introduced into the model to test their potential bias to the main results. The dlnm and INLA packages in R software, version 4.1.3 (R Foundation for Statistical Computing) were used for building models.^35,39^ Data were analyzed in May 2022.

## Results

### Descriptive Analysis

In total, 70 006 dengue fever cases were reported from 54 cities in the 4 provinces. Dengue fever incidence data showed obvious seasonality and interannual variation, with the majority of cases reported from May to November. An epidemic occurred in 2014 and 2019 (eFigures 3 and 4 in Supplement 1). The meteorological and hydrological conditions of the 4 provinces showed spatiotemporal heterogeneity (eFigures 5 and 6 in Supplement 1).

### Association Between Hydrological Conditions and Dengue Fever 

**Figure 3.**
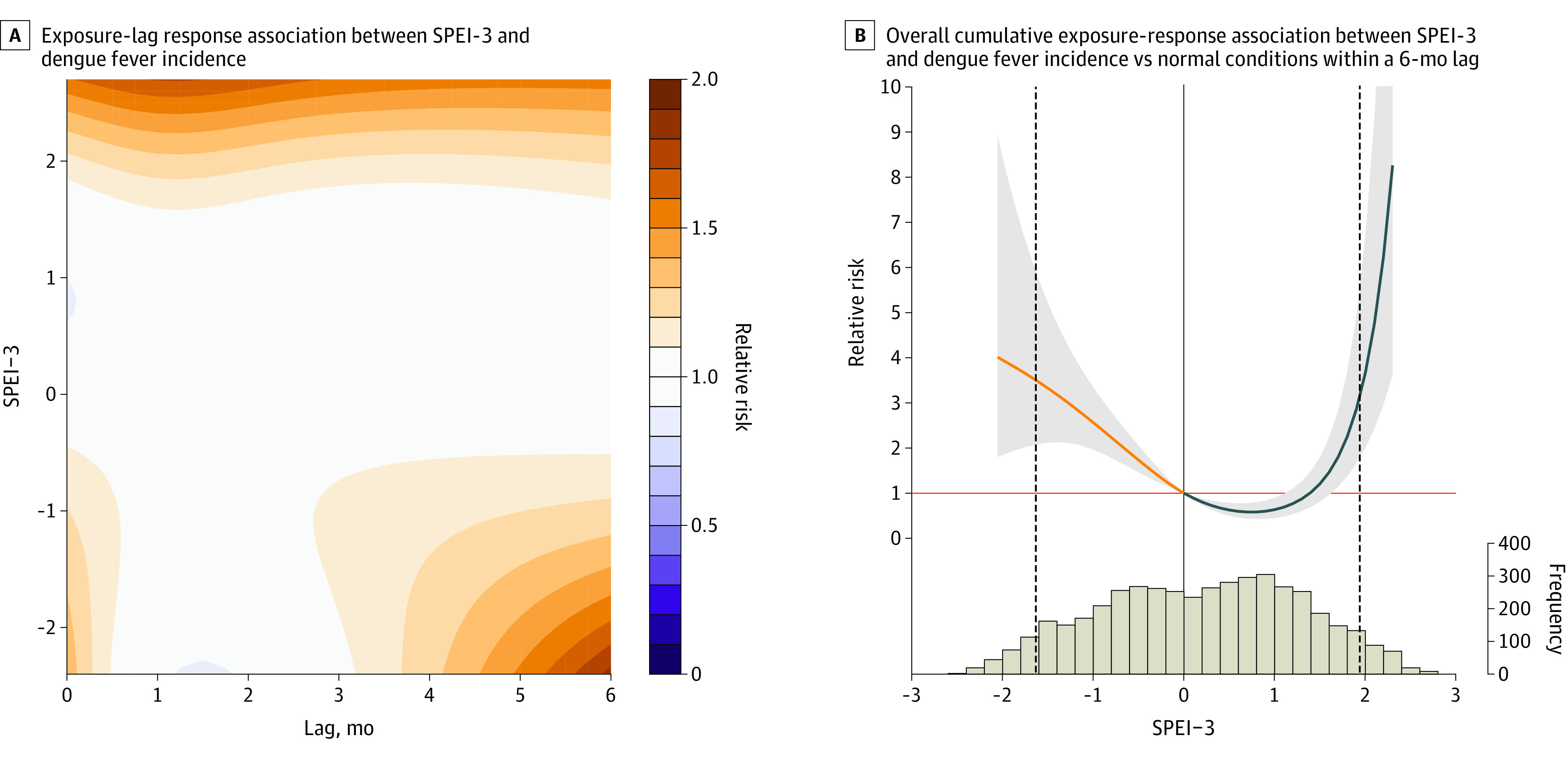
Relative Risk of Dengue Fever With Standardized Precipitation Evapotranspiration Index (SPEI)–3 Exposures and Lags in the 4 Provinces in China In panel B, the gray shading represents the 95% credible interval. The solid vertical line indicates the SPEI-3 equal to 0, and the dashed vertical lines represent the 2.5 and 97.5 percentiles of SPEI-3.

Extreme wet conditions were associated with a high RR of dengue fever incidence throughout the lag period, with the effect size peaking at the 1-month lag (RR, 1.27; 95% CrI, 1.05-1.53). In comparison, the effect size of extreme dry conditions had a longer delay (4- to 6-month lag), with the highest effect size occurring at the 6-month lag (RR, 1.63; 95% CrI, 1.29-2.05). Overall, a U-shaped association was observed between SPEI-3 and dengue fever incidence over a 6-month lag. The cumulative RRs were 3.66 (95% CrI, 2.00-6.70) for extreme wet conditions and 3.94 (95% CrI, 1.85-8.42) for extreme dry conditions (Figure 3). The RR of dengue fever increased significantly when the monthly minimum temperature was higher than the median value (18.2 °C), and the cumulative RR was the highest when the monthly minimum temperature was 25.5 °C (eFigure 7 in Supplement 1). Sensitivity analyses showed that the main results changed little when changing the parameters and meteorological variables in the model (eFigure 8 in Supplement 1).

### Modification Role of City Development

**Figure 4.**
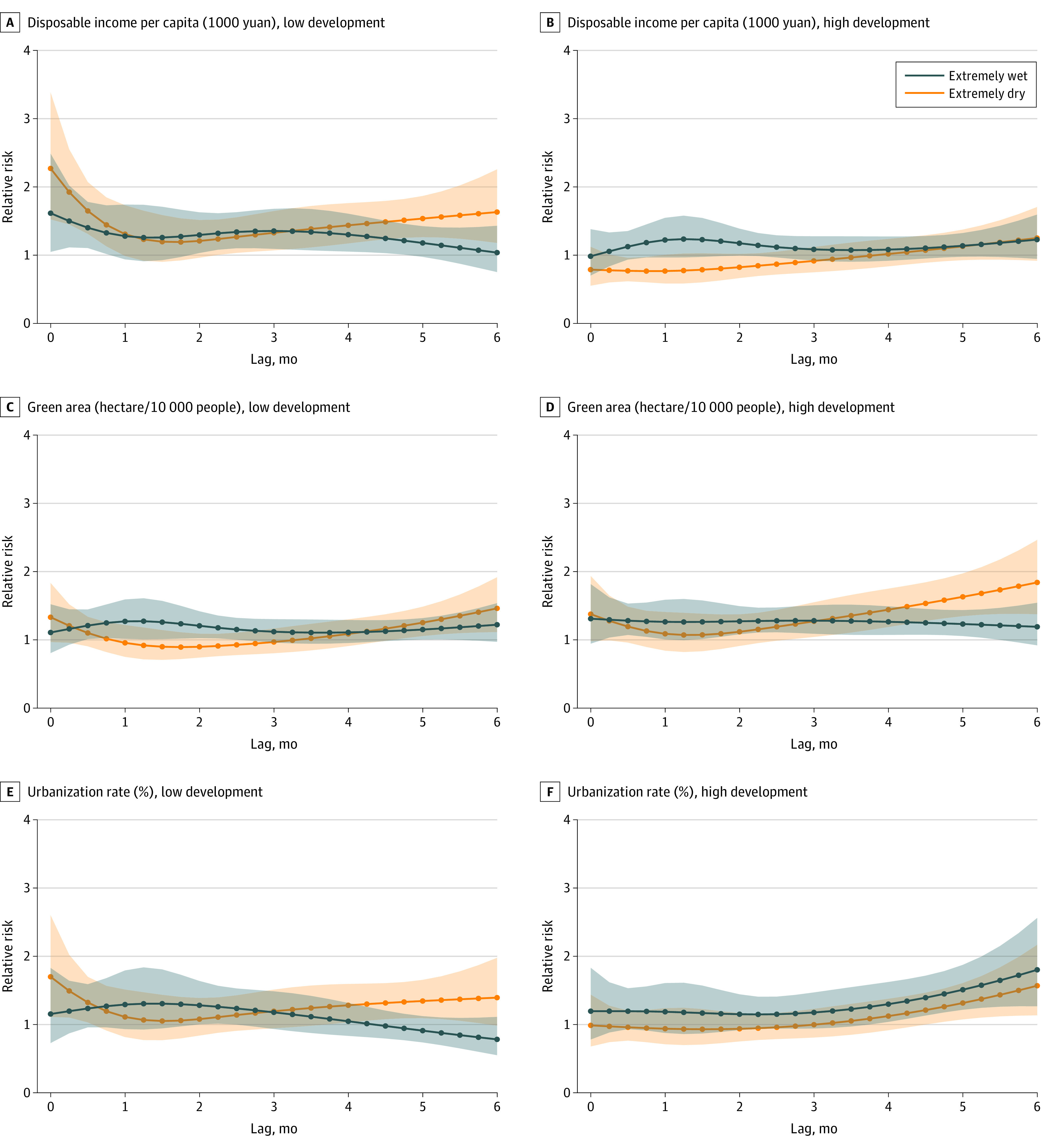
Lag-Response Associations for Extreme Wet and Extreme Dry Conditions Under High- and Low-Development Scenarios Extreme wet conditions had a Standardized Precipitation Evapotranspiration Index (SPEI)–3 of 2, and extreme dry conditions had an SPEI-3 of −2. To convert yuan to US dollars, multiply by 0.14.

City economic development (GDP per capita), public services (number of physicians), residents’ quality of life (disposable income), ecological environment (green area), and city building (urbanization rate) had various modification roles in the association between hydrological conditions and dengue fever incidence (Figure 4; Table; eFigures 9 and 10 in Supplement 1). Economic development, public services, and residents’ quality of life played similar modification roles in the association. Higher development levels were factors in reduced adverse implications of hydrological extremes, especially extreme dry conditions. Specifically, in areas with lower GDP per capita, number of physicians, and disposable income, the risk of dengue fever immediately increased after extreme dry conditions, with the largest RRs observed at a 0-month lag (lower GDP per capita: 1.76 [95% CrI, 1.21-2.57]; number of physicians: 2.04 [95% CrI, 1.40-2.98]; and disposable income: 2.27 [95% CrI, 1.52-3.38]) and the cumulative RRs found within 6 months (lower GDP per capita: 7.15 [95% CrI, 2.89-17.69]; number of physicians: 11.81 [95% CrI, 5.16-27.03]; and disposable income: 17.10 [95% CrI, 7.17-40.78]). Similar and comparatively modest modification patterns were found under extreme wet conditions.

**Table.** Maximum and Cumulative Relative Risk of Dengue Fever for Extreme Wet and Extreme Dry Conditions Within 6 Months by Different Scenarios of City Development Indicators

City development indicator	Extreme wet conditions: SPEI-3 of 2			Extreme dry conditions: SPEI-3 of −2		
	Maximum RR and lag		Cumulative RR (95% CrI)	Maximum RR and lag		Cumulative RR (95% CrI)
	RR (95% CrI)	Lag, mo		RR (95% CrI)	Lag, mo	
**GDP per capita**						
Low	1.41 (0.95-2.11)	0	4.47 (1.84-10.87)	1.76 (1.21-2.57)	0	7.15 (2.89-17.69)
Medium	1.28 (0.95-1.74)	0	4.00 (2.08-7.71)	1.52 (1.12-2.06)	0	4.61 (2.16-9.87)
High	1.30 (1.00-1.68)	1	2.73 (1.15-6.48)	1.67 (1.21-2.30)	6	1.69 (0.53-5.36)
**No. of physicians per 10 000 people**						
Low	1.62 (1.07-2.44)	0	5.56 (2.19-14.11)	2.04 (1.40-2.98)	0	11.81 (5.16-27.03)
Medium	1.38 (1.00-1.89)	0	4.29 (2.14-8.58)	1.60 (1.18-2.16)	0	4.90 (2.28-10.53)
High	1.21 (0.99-1.48)	2	2.16 (0.97-4.82)	1.27 (0.92-1.75)	6	0.82 (0.28-2.39)
**Disposable income per capita**						
Low	1.61 (1.05-2.49)	0	5.74 (2.18-15.14)	2.27 (1.52-3.38)	0	17.10 (7.17-40.78)
Medium	1.40 (1.01-1.93)	0	4.46 (2.22-8.96)	1.74 (1.27-2.39)	0	5.83 (2.73-12.44)
High	1.24 (0.97-1.58)	1	2.31 (1.02-5.24)	1.25 (0.92-1.70)	6	0.65 (0.23-1.89)
**Green area per 10 000 people**						
Low	1.27 (1.00-1.61)	1	2.91 (1.43-5.90)	1.46 (1.11-1.91)	6	2.16 (0.91-5.10)
Medium	1.27 (1.02-1.59)	1	3.15 (1.64-6.07)	1.52 (1.18-1.96)	6	2.65 (1.19-5.90)
High	1.31 (0.94-1.81)	0	4.92 (2.20-10.98)	1.84 (1.37-2.46)	6	9.08 (3.17-26.00)
**Urbanization rate**						
Low	1.30 (0.94-1.80)	2	1.65 (0.57-4.73)	1.70 (1.11-2.60)	0	5.74 (2.36-13.98)
Medium	1.29 (1.03-1.63)	1	2.90 (1.51-5.58)	1.51 (1.09-2.08)	0	3.92 (1.81-8.49)
High	1.80 (1.26-2.56)	6	6.67 (2.16-20.62)	1.57 (1.13-2.16)	6	1.97 (0.69-5.60)

Abbreviations: CrI, credible interval; GDP, gross domestic product; RR, relative risk; SPEI, Standardized Precipitation Evapotranspiration Index.

In terms of the modification role of the ecological environment, areas with larger green space per capita were more vulnerable to dengue fever after extreme dry conditions. Compared with areas with limited green space, the risk of dengue fever in areas with rich green space increased 3 to 6 months after extreme dry conditions, with the maximum single-month RR equating to 1.84 (95% CrI, 1.37-2.46) at a 6-month lag and the cumulative RR equating to 9.08 (95% CrI, 3.17-26.00).

The urbanization rate had an adverse modification role in extreme wet conditions but a beneficial modification role in extreme dry conditions. Areas with lower urbanization rates had the highest risk of dengue fever in extreme dry months (RR, 1.70; 95% CrI, 1.11-2.60). Highly urbanized areas had a higher risk of dengue fever 4 to 6 months after extreme wet conditions, with the maximum single-month RR equating to 1.80 (95% CrI, 1.26-2.56) at a 6-month lag.

## Discussion

Similar to other studies,^40,41^ we found that extreme dry and extreme wet conditions were both associated with increased dengue fever risk. We also found that the cumulative effect size of wet conditions was higher than that of dry conditions, supporting the findings from a previous study^42^ in Sri Lanka that the wet zone was at a greater risk than the dry zone. The findings of the present study are believed to be the response and adaptive behavior of both the vector and host to different hydrological extremes. On the one hand, precipitation has been known to trigger the hatching process of outdoor *Aedes aegypti* eggs.^43,44^ Previous studies^45,46,47^ have found an association between an increased abundance of *Aedes aegypti* and precipitation. On the other hand, dry conditions were associated with an increased dengue fever risk in a longer lag period. This result was consistent with the findings of a study^32^ in Barbados that found that dengue fever incidence was greatest 1 to 2 months after exceptionally wet conditions, and exceptionally dry conditions were associated with increased dengue fever risk 5 months later. This finding could be explained by the change in water storage practices during a protracted severe drought.^48^ Given that female *Aedes albopictus* mosquitoes usually breed in small artificial containers around human dwellings, the seasonal pattern of mosquito population dynamics may be more affected by variations in human water supply than changes in precipitation.^49^ People tend to use improvised artificial water storage containers to cope with the absence of precipitation, which increases the breeding locations for *Aedes albopictus*.^50^ Therefore, people should be warned of the risk of using artificial water storage containers, especially in the context of climate change and associated intense and frequent extreme precipitation and severe drought.^51^

Findings of this study suggested that indicators of the local economic level, health care resources, and residents’ quality of life had an essential modification role in the association between hydrological conditions and dengue fever incidence. Dengue fever incidence was higher in the low-development scenarios. First, people living in areas with a low economic level may be less adaptable to hydrological extremes, such as the lack of mosquito prevention equipment in their houses. Second, poor sanitation, including limited water supply, may encourage mosquito breeding through the use of uncovered water storage containers.^12^ In addition, the lack of high-quality health care services in underdevelopment areas may delay the detection and treatment of dengue fever cases.^52^ This result has implications for public health services. For example, there should be a mosquito prevention infrastructure near residential areas, sustainable clean water supply, and rational arrangement of health care resources to help residents cope with potential dengue fever outbreaks, which may enhance dengue fever prevention, detection, and treatment capacity in undeveloped areas.

This study found that cities with large green areas had a higher dengue fever risk in extreme dry conditions, with the maximum risk occurring after a 6-month delay. Previous studies^53,54^ have reported that green spaces in the city may attract adult *Aedes aegypti* and become water reservoirs for laying eggs after a long-term drought. Several studies^55,56,57^ have indicated the important role of greenness in sustaining mosquito populations. In extreme dry conditions, open containers in public green areas likely serve as incubators for mosquito eggs and larvae. Furthermore, garden watering may be a substitute for natural precipitation as the primary factor in egg incubation in summers with limited precipitation.^49,58^ One suggestion is to plan green areas and densely populated areas during city construction to reduce vector-host contact. Additionally, mosquito control activities in green areas could be bolstered by eliminating potential mosquito habitats.

Urbanization was another modification factor in the association between hydrological conditions and dengue fever incidence. In this study, an immediate risk of dengue fever under extreme dry conditions was observed only in less urbanized regions. The urbanization rate is usually associated with advanced piped water networks^12^; therefore, the risk of dengue fever in highly urbanized areas is expected not to increase significantly after extreme dry conditions compared with rural areas. On the contrary, after a long-term extreme drought, the increased use of water storage containers and going outside to fetch water boost the likelihood of mosquito breeding and encounters in less developed areas.^54^ In addition, we observed that the dengue fever risk was higher in highly urbanized regions after 3 months of extreme wet conditions. Heavy precipitation may destroy some of the existing habitats of *Aedes aegypti* larvae, but more importantly, abundant new spawning sites will become available subsequently. Highly urbanized areas may have more outdoor breeding sites, such as rain-filled waste and garbage containers.^12,59^ Hence, the risk of dengue fever is likely to increase after the whole development cycle of *Aedes aegypti* and the incubation period of dengue virus.^13^ Conversely, a study^60^ found that the collection of household garbage and tires with water-retention capacity was associated with lower dengue fever incidence in Recife, Brazil. However, field surveillance is required to collect mosquito and habitat data to test our hypotheses.

### Limitations

The limitations of this study should be acknowledged. First, meteorological data were collected from the National Climate Center of China Meteorological Administration at a coarse resolution, which may not fully capture the spatial difference across cities and thus reduce the magnitude of SPEI–dengue fever incidence association. This issue may be mitigated in the future when higher-resolution data sets are developed for the study areas. Second, the lack of high-quality vector density data may diminish understanding of the mechanisms of the association between hydrological patterns and dengue fever incidence. Third, the spatial connection pattern of the model assumed that connectivity existed only between adjacent cities. However, the mobility of people may go beyond this limit in the real world. Future studies are encouraged to depict the complex population mobility. Fourth, city-level socioeconomic data were used, which cannot account for the exposure variation across subregions and individuals within the city. Small-scale socioeconomic data are suggested in further research.^52^

## Conclusions

This cross-sectional study found that extreme hydrological conditions were associated with higher dengue fever incidence within a 6-month lag period, and these associations were more noticeable in underdeveloped and highly green cities. Regional disparities in city development played a role in exacerbating the adverse implications of hydrological conditions for dengue fever incidence and compromising population health. These findings highlight the need to develop climate change adaptation strategies and public health interventions in regions vulnerable to dengue fever.

## Notes

Supplement 1. **eMethods.** Computation of Standardized Precipitation Evapotranspiration Index **eTable 1.** Fifty Indicators for Evaluating Quality of City Development **eTable 2.** Description of 12 City Development Indicators in the Four Provinces in China, 2013-2019 [Median (P25th, P75th)] **eTable 3.** Spearman Correlation Coefficients of 12 City Development Indicators in the Four Provinces in China, 2013-2019 **eTable 4.** Model Goodness-of-Fit Statistics Results **eFigure 1.** Spatial Distribution of Annual Mean Value of City Development Indicators for the 54 Cities of the Four Provinces in China, 2013-2019 **eFigure 2.** The Flowchart of Model Construction Procedure **eFigure 3.** The Annual Dengue Incidence in the Four Provinces in China, 2013-2019 **eFigure 4.** Monthly Dengue Incidence Rates (per 10 000 000 Population) at Provincial Level, 2013-2019 **eFigure 5.** Monthly SPEI-3, -6, and -12 at Provincial Level, 2013-2019 **eFigure 6.** Monthly T_mean_, T_max_, T_min_ (°C), and Precipitation (mm) at Provincial Level, 2013-2019 **eFigure 7.** Relative Risk of Dengue With T_min_ Exposures and Lags in the Four Provinces in China **eFigure 8.** Sensitivity Results of Cumulative Relative Risk of SPEI-3 on Dengue Incidence **eFigure 9.** Lag-Response Associations for Extreme Wet and Extreme Dry Conditions Under High- and Low-Economic and Public Services Development Scenario **eFigure 10.** Contour Plots of the Exposure-Lag-Response Associations Between the SPEI-3 and Dengue Incidence Overall and Under High-, Middle-, and Low-Development Scenario
eReferences

Supplement 2.
Data Sharing Statement
