# Supplementary material for: Association Between Hydrological Conditions and Dengue Fever Incidence in Coastal Southeastern China From 2013 to 2019
Source: JAMA Netw Open. 2023 Jan 4;6(1):e2249440. doi: 10.1001/jamanetworkopen.2022.49440 (PMC9857674; doi:10.1001/jamanetworkopen.2022.49440)
Supplement: Supplement 1. — eMethods. Computation of Standardized Precipitation Evapotranspiration Index eTable 1. Fifty Indicators for Evaluating Quality of City Development eTable 2. Description of 12 City Development Indicators in the Four Provinces in China, 2013-2019 [Median (P25th, P75th)] eTable 3. Spearman Correlation Coefficients of 12 City Development Indicators in the Four Provinces in China, 2013-2019 eTable 4. Model Goodness-of-Fit Statistics Results eFigure 1. Spatial Distribution of Annual Mean Value of City Development Indicators for the 54 Cities of the Four Provinces in China, 2013-2019 eFigure 2. The Flowchart of Model Construction Procedure eFigure 3. The Annual Dengue Incidence in the Four Provinces in China, 2013-2019 eFigure 4. Monthly Dengue Incidence Rates (per 10 000 000 Population) at Provincial Level, 2013-2019 eFigure 5. Monthly SPEI-3, -6, and -12 at Provincial Level, 2013-2019 eFigure 6. Monthly Tmean, Tmax, Tmin (°C), and Precipitation (mm) at Provincial Level, 2013-2019 eFigure 7. Relative Risk of Dengue With Tmin Exposures and Lags in the Four Provinces in China eFigure 8. Sensitivity Results of Cumulative Relative Risk of SPEI-3 on Dengue Incidence eFigure 9. Lag-Response Associations for Extreme Wet and Extreme Dry Conditions Under High- and Low-Economic and Public Services Development Scenario eFigure 10. Contour Plots of the Exposure-Lag-Response Associations Between the SPEI-3 and Dengue Incidence Overall and Under High-, Middle-, and Low-Development Scenario eReferences [file jamanetwopen-e2249440-s001.pdf]

# Supplemental Online Content

Li C, Wang Z, Yan Y, et al. Association between hydrological conditions and dengue incidence in coastal southeastern China from 2013 to 2019. *JAMA Netw Open*. 2023;6(1):e2249440. doi:10.1001/jamanetworkopen.2022.49440

**eMethods.** Computation of Standardized Precipitation Evapotranspiration Index
**eTable 1.** Fifty Indicators for Evaluating Quality of City Development
**eTable 2.** Description of 12 City Development Indicators in the Four Provinces in China, 2013-2019 [Median (P25th, P75th)]
**eTable 3.** Spearman Correlation Coefficients of 12 City Development Indicators in the Four Provinces in China, 2013-2019
**eTable 4.** Model Goodness-of-Fit Statistics Results
**eFigure 1.** Spatial Distribution of Annual Mean Value of City Development Indicators for the 54 Cities of the Four Provinces in China, 2013-2019
**eFigure 2.** The Flowchart of Model Construction Procedure
**eFigure 3.** The Annual Dengue Incidence in the Four Provinces in China, 2013-2019
**eFigure 4.** Monthly Dengue Incidence Rates (per 10 000 000 Population) at Provincial Level, 2013-2019
**eFigure 5.** Monthly SPEI-3, -6, and -12 at Provincial Level, 2013-2019
**eFigure 6.** Monthly $T_{mean}$, $T_{max}$, $T_{min}$ (˚C), and Precipitation (mm) at Provincial Level, 2013-2019
**eFigure 7.** Relative Risk of Dengue With $T_{min}$ Exposures and Lags in the Four Provinces in China
**eFigure 8.** Sensitivity Results of Cumulative Relative Risk of SPEI-3 on Dengue Incidence
**eFigure 9.** Lag-Response Associations for Extreme Wet and Extreme Dry Conditions Under High- and Low-Economic and Public Services Development Scenario
**eFigure 10.** Contour Plots of the Exposure-Lag-Response Associations Between the SPEI-3 and Dengue Incidence Overall and Under High-, Middle-, and Low-Development Scenario
**eReferences**

This supplemental material has been provided by the authors to give readers additional information about their work.

© 2023 Li C et al. *JAMA Network Open.*

## eMethods. Computation of standardized precipitation evapotranspiration index

Many hydrological indicators have been developed to monitor the characteristic of drought episodes in terms of the severity, duration and spatial extent, such as standardized precipitation index (SPI), standardized precipitation evapotranspiration index (SPEI), and Palmer drought severity index (PDSI).[1-4] It is generally accepted that drought is a multi-scale phenomenon, hence SPI allows to be calculated at different time scales to monitor droughts with respect to different usable water resources, and has become the most widely used drought index globally.

The precipitation-based drought indices including SPI assume that the variability of precipitation is much higher than other variables such as temperature and potential evapotranspiration (PET), and drought is only controlled by the temporal variability in precipitation. However, a major criticism is that the importance of the impact of temperature on drought conditions should not be ignored.[5,6] Therefore, the SPEI was developed based on precipitation and PET data. The SPEI combines the simplicity of calculation and the multi-temporal character of the SPI with the sensitivity of PDSI to evaporation demand variations (caused by temperature fluctuations and trends).[2,7] As a result, SPEI can be considered as a more preferable indicator in this regard.[4,8]

The SPEI uses the monthly difference between precipitation and PET. This represents a simple climatic water balance which is calculated at different time scales to obtain the SPEI. The first and key step in calculating the SPEI is to effectively estimate the PET, In this study, we calculated the monthly PET series of 54 cities in the four provinces during 1960-2019 using Thornthwaite approach:[9]

$$PET = 16 \times (\frac{10T_i}{H})^A$$

where $T_i$ denotes monthly mean temperature (°C), $H$ denotes the annual cumulative heat index derived by summing the monthly heat values ($H_i$) of the year. $A$ is a $H$-related constant. $H$, $H_i$, and $A$ can be derived according to the following formulas:

$$H_i = (\frac{T_i}{5})^{1.514}$$

$$H = \sum_{i=1}^{12} H_i$$

$$A = 6.75 \times 10^{-7}H^3 - 7.71 \times 10^{-5}H^2 + 1.792 \times 10^{-2}H + 0.49$$

Secondly, we calculated the difference ($D_i$) between actual precipitation ($P_i$) and $PET_i$ of each city for the month i:

$$D_i = P_i - PET_i$$

Thirdly, we normalized the $D_i$ series and calculated the SPEI corresponding to each value. Note that SPEI uses the log-logistic probability distribution with three parameters to address the possible negative values in $D_i$ series. The probability distribution function is expressed as:

$$F_{(x)} = [1 + (\frac{\alpha}{x-\gamma})^{\beta}]^{-1}$$

where α, β and γ are scale, shape and origin parameters, respectively, and calculated by using L-moment approach:[10]

$$\alpha = \frac{(W_0 - 2W_1)\beta}{\Gamma(1+\frac{1}{\beta})\Gamma(1-\frac{1}{\beta})}$$

$$\beta = \frac{(2W_1 - W_0)}{(6W_1 - W_0 - 6W_2)}$$

$$\gamma = W_0 - \alpha\Gamma(1+\frac{1}{\beta})\Gamma(1-\frac{1}{\beta})$$

We used probability weighted moments method to calculate the log-logistic α, β and γ distribution parameters, where the PWMs of order s ($W_s$) were calculated as:

$$W_s = \frac{1}{N}\sum_{i=1}^{N}(1-F_i)^s D_i$$

$$F_i = \frac{i - 0.35}{N}$$

where $N$ is the number of data, $F_i$ is a frequency estimator following the approach of Hosking[11] for the month i.

© 2023 Li C et al. *JAMA Network Open.*

Finally, for cumulative probability ($P$) lower than 0.5, $P = 1 - F_{(x)}$, and SPEI can be obtained as:

$$SPEI = W - \frac{c_0 - c_1 W + c_2 W^2}{1 + d_1 W + d_2 W^2 + d_3 W^3}$$

where $c_0$= 2.515517, $c_1$= 0.802853, $c_2$= 0.010328, $d_1$= 1.432788, $d_2$= 0.189269, $d_3$ = 0.001308, and $W = \sqrt{-2\ln{(P)}}$.

For $P$ > 0.5, $P = 1 - P$ and the SPEI was calculated as:

$$SPEI = -(W - \frac{c_0 - c_1 W + c_2 W^2}{1 + d_1 W + d_2 W^2 + d_3 W^3})$$

© 2023 Li C et al. *JAMA Network Open.*

**eTable 1. Fifty indicators for evaluating quality of city development**

| Indicator | Unit |
|---|---|
| 1. Economic development | |
| 1.1 GDP per capita[a] | 10 000 yuan/people |
| 1.2 Total labor productivity | 10 000 yuan/people |
| 1.3 Proportion of research and experimental development (R&D) expenditure in GDP | % |
| 1.4 Number of invention patents per billion yuan of GDP | /1 000 000 000 yuan |
| 1.5 Proportion of technology contract turnover in GDP | % |
| 1.6 Number of national standards formulated and revised per 100 000 people | /100 000 people |
| 1.7 Number of China Quality Award | - |
| 1.8 Number of high-tech enterprises per 10 000 enterprise legal persons | - |
| 1.9 Number of market entities per 100 000 people | - |
| 1.10 Amount of foreign capital actually utilized per capita | USD/people |
| 1.11 Total trade in goods and services per capita | 10 000 yuan/people |
| 2. Ecological environment | |
| 2.1 Average annual concentration of PM 2.5[a] | $\mu g/m^3$ |
| 2.2 Ratio of days with good or moderate air quality | % |
| 2.3 The proportion of surface water reaching or better than Class III | % |
| 2.4 Energy consumption per 10 000-yuan GDP | tce/10 000 yuan |
| 2.5 Carbon emission per 10 000-yuan GDP | $tCO_2e$/10 000 yuan |
| 2.6 Number of green factories per 100 industry enterprises above Designated Size | /100 industry enterprises |
| 2.7 Emission intensity of main air pollutants from fixed pollution sources | /10 000 yuan |
| 2.8 Discharge intensity of main water pollutants from fixed pollution sources | /10 000 yuan |
| 2.9 Comprehensive utilization rate of general industrial solid waste | % |
| 2.10 Green area per 100 000 people[a,b] | $hm^2$/100 000 people |
| 2.11 Forest coverage[a] | % |
| 3. City building | |
| 3.1 Construction land use area per 10 000-yuan GDP | $km^2$/10 000 yuan |
| 3.2 Urbanization rate of permanent residents[a,c] | % |
| 3.3 Number of buses per 10 000 people[a] | /10 000 people |
| 3.4 Kilometers of rail transit system per 100 000 people | km/100 000 people |
| 3.5 Road area per capita[a] | $m^2$/people |
| 3.6 Ratio of cars to parking spaces | - |

© 2023 Li C et al. *JAMA Network Open.*

| Indicator | Unit |
|---|---|
| 3.7 Park area per capita | $hm^2$/people |
| 3.8 Density of drainage pipeline in built-up area | $km/km^2$ |
| 3.9 Download rate of broadband users | Mbps |
| 4. Public services | |
| 4.1 Expenditure on public services per 10 000 people | 10 000 yuan/10 000 people |
| 4.2 Duration of business establishment | days |
| 4.3 Education expenditure per student | 10 000 yuan/people |
| 4.4 Ratio of teachers and students in primary and secondary schools[a] | - |
| 4.5 Number of physicians per 1000 people[a] | /1000 people |
| 4.6 Number of nurses per 1000 people | /1000 people |
| 4.7 Number of beds in medical institutions per 1000 people[a] | /1000 people |
| 4.8 Number of nursing beds per 1000 elderly people | /1000 elderly people |
| 4.9 Number of deaths due to production safety accidents per 100 000 000-yuan GDP | /100 000 000 yuan |
| 5. Life quality | |
| 5.1 Unemployment rate | % |
| 5.2 Disposable income per capita[a] | 10 000 yuan |
| 5.3 Consumption expenditure per capita[a] | 10 000 yuan |
| 5.4 Engel coefficient | % |
| 5.5 Proportion of personal health expenditure in total health expenditure | % |
| 5.6 Life expectancy | years |
| 5.7 Amount of basic endowment insurance | yuan/(month·people) |
| 5.8 Years of education of working age population | years |
| 5.9 Housing area per capita | $m^2$/people |
| 5.10 Number of cultural center services per capita | - |

Note: These 50 variables are standard indicators recommended by China’s State Administration for Market Regulation to reflect the quality of city development. To convert yuan to US dollars, multiply by 0.14.
[a] These 12 variables are preliminarily selected in this study.
[b] Green area refers to the total area of all green spaces in a city, including public areas, roads areas, residential areas, institutional lands, protection areas, production areas, and scenic areas.
[c] Urbanization rate refers to the percentage of the number of permanent residents in urban area at the end of a year to the total number of permanent residents at the end of the year.

© 2023 Li C et al. *JAMA Network Open.*

**eTable 2. Description of 12 city development indicators in the four provinces in China, 2013-2019 [Median (P25th, P75th)]**

| Indicators | Zhejiang | Fujian | Guangdong | Guangxi |
|---|---|---|---|---|
| Economic development | | | | |
| GDP per capita (1000 yuan) | 72.66 (58.85, 97.43) | 70.00 (58.99, 89.34) | 42.85 (32.27, 86.04) | 33.94 (25.81, 49.07) |
| Ecological environment | | | | |
| PM 2.5 concentration (μg/m$^3$) | 28.50 (23.30, 34.57) | 22.83 (19.22, 26.82) | 25.30 (21.46, 30.42) | 25.99 (23.39, 29.17) |
| Green area (hectare/10 000 people) | 11.97 (8.75, 15.70) | 7.85 (5.55, 13.28) | 11.81 (6.13, 25.58) | 7.69 (5.24, 15.81) |
| Forest coverage (%) | 60.65 (42.70, 68.80) | 59.50 (50.30, 64.10) | 46.80 (35.20, 67.20) | 64.50 (52.60, 74.10) |
| City building | | | | |
| Urbanization rate (%) | 63.60 (59.70, 69.00) | 57.70 (54.80, 65.90) | 55.20 (47.79, 86.38) | 46.57 (38.95, 54.46) |
| Number of buses (/10 000 people) | 2.58 (1.77, 4.43) | 1.63 (1.29, 3.95) | 1.93 (1.00, 7.40) | 1.27 (0.48, 2.46) |
| Road area per capita (m$^2$) | 4.65 (3.16, 5.73) | 2.67 (1.66, 4.31) | 2.95 (1.77, 5.45) | 2.92 (1.89, 5.15) |
| Public service | | | | |
| Ratio of students to teachers in primary and secondary schools | 14.93 (14.29, 15.74) | 14.68 (13.51, 16.59) | 15.93 (14.9, 17.02) | 17.75 (16.91, 18.62) |
| Number of hospital beds (per 10 000 people) | 45.03 (40.79, 53.19) | 41.17 (37.33, 48.80) | 33.51 (28.80, 40.69) | 36.08 (30.71, 42.67) |
| Number of physicians (per 10 000 people) | 29.11 (25.99, 32.26) | 20.00 (18.15, 23.24) | 19.38 (17.02, 23.25) | 17.92 (15.72, 21.72) |
| Life quality | | | | |
| Disposable income per capita (1000 yuan) | 46.50 (39.76, 54.44) | 33.02 (27.82, 38.98) | 27.30 (22.91, 38.32) | 28.36 (25.04, 32.16) |
| Consumption expenditure per capita (1000 yuan) | 29.12 (25.30, 33.32) | 22.36 (19.19, 26.39) | 19.35 (16.06, 26.64) | 17.45 (15.65, 19.36) |

To convert yuan to US dollars, multiply by 0.14.

© 2023 Li C et al. *JAMA Network Open.*

**eTable 3. Spearman correlation coefficients of 12 city development indicators in the four provinces in China, 2013-2019**

| | GDP | PM 2.5 | Green | Forest | Urban | Bus | Road | RST | Bed | Physician | Income | Expend |
|---|---|---|---|---|---|---|---|---|---|---|---|---|
| GDP | 1.000 | -0.049 | 0.449 | -0.130 | 0.580 | 0.576 | 0.434 | -0.340 | 0.344 | 0.564 | 0.538 | 0.597 |
| PM 2.5 | -0.049 | 1.000 | 0.241 | -0.353 | 0.217 | 0.193 | 0.257 | 0.252 | 0.014 | -0.016 | 0.070 | 0.005 |
| Green | 0.449 | 0.241 | 1.000 | -0.400 | 0.679 | 0.759 | 0.858 | 0.203 | 0.273 | 0.542 | 0.565 | 0.572 |
| Forest | -0.130 | -0.353 | -0.400 | 1.000 | -0.537 | -0.428 | -0.399 | -0.184 | 0.030 | -0.085 | -0.334 | -0.378 |
| Urban | 0.580 | 0.217 | 0.679 | -0.537 | 1.000 | 0.825 | 0.702 | 0.031 | 0.291 | 0.627 | 0.728 | 0.799 |
| Bus | 0.576 | 0.193 | 0.759 | -0.428 | 0.825 | 1.000 | 0.774 | 0.033 | 0.369 | 0.689 | 0.685 | 0.725 |
| Road | 0.434 | 0.257 | 0.858 | -0.399 | 0.702 | 0.774 | 1.000 | 0.267 | 0.295 | 0.616 | 0.675 | 0.661 |
| RST | -0.340 | 0.252 | 0.203 | -0.184 | 0.031 | 0.033 | 0.267 | 1.000 | -0.253 | -0.137 | 0.036 | -0.042 |
| Bed | 0.344 | 0.014 | 0.273 | 0.030 | 0.291 | 0.369 | 0.295 | -0.253 | 1.000 | 0.538 | 0.420 | 0.403 |
| Physician | 0.564 | -0.016 | 0.542 | -0.085 | 0.627 | 0.689 | 0.616 | -0.137 | 0.538 | 1.000 | 0.755 | 0.755 |
| Income | 0.538 | 0.070 | 0.565 | -0.334 | 0.728 | 0.685 | 0.675 | 0.036 | 0.420 | 0.755 | 1.000 | 0.951 |
| Expend | 0.597 | 0.005 | 0.572 | -0.378 | 0.799 | 0.725 | 0.661 | -0.042 | 0.403 | 0.755 | 0.951 | 1.000 |

Note: GDP, GDP per capita (1000 yuan); PM 2.5, PM 2.5 concentration (μg/$m^3$); Green, Green area (hectare/10 000 people); Forest, Forest coverage (%); Urban, Urbanization rate (%), Bus, Number of bus (/10 000 people); Road, Road area per capita ($m^2$); RST, Ratio of students to teachers in primary and secondary schools; Bed, Number of hospital beds (per 10 000 people); Physician, Number of physicians (per 10 000 people); Income, Disposable income per capita (1000 yuan); Expend, Consumption expenditure per capita (1000 yuan). To convert yuan to US dollars, multiply by 0.14.

© 2023 Li C et al. *JAMA Network Open.*

## eTable 4. Model goodness-of-fit statistics results

| Model | Equation | DIC | CV log score |
|---|---|---|---|
| Baseline model | $\log\left(E(Y_{ct})\right) = \alpha + \beta_{p(p)m(t)} + \varphi_{sy(t)} + \upsilon_{sy(t)}$ | 10892.90 | 1.349 |
| Hydrological model | | | |
| Baseline model + SPEI-3 | $\log\left(E(Y_{ct})\right) = \alpha + \beta_{p(p)m(t)} + \varphi_{sy(t)} + \upsilon_{sy(t)} + cb(SPEI\!-3, l)$ | 10839.69 | 1.350 |
| Baseline model + SPEI-6 | $\log\left(E(Y_{ct})\right) = \alpha + \beta_{p(p)m(t)} + \varphi_{sy(t)} + \upsilon_{sy(t)} + cb(SPEI\!-6, l)$ | 10851.88 | 1.358 |
| Baseline model + SPEI-12 | $\log\left(E(Y_{ct})\right) = \alpha + \beta_{p(p)m(t)} + \varphi_{sy(t)} + \upsilon_{sy(t)} + cb(SPEI\!-12, l)$ | 10874.27 | 1.353 |
| Hydrological and meteorological model | | | |
| Baseline model + SPEI-3 + $T_{mean}$ | $\log\left(E(Y_{ct})\right) = \alpha + \beta_{p(p)m(t)} + \varphi_{sy(t)} + \upsilon_{sy(t)} + cb(SPEI\!-3, l) + cb(T_{mean}, l)$ | 10815.80 | 1.351 |
| Baseline model + SPEI-3 + $T_{max}$ | $\log\left(E(Y_{ct})\right) = \alpha + \beta_{p(p)m(t)} + \varphi_{sy(t)} + \upsilon_{sy(t)} + cb(SPEI\!-3, l) + cb(T_{max}, l)$ | 10825.59 | 1.342 |
| Baseline model + SPEI-3 + $T_{min}$ | $\log\left(E(Y_{ct})\right) = \alpha + \beta_{p(p)m(t)} + \varphi_{sy(t)} + \upsilon_{sy(t)} + cb(SPEI\!-3, l) + cb(T_{min}, l)$ | 10808.74 | 1.348 |
| Baseline model + SPEI-3 + Pre | $\log\left(E(Y_{ct})\right) = \alpha + \beta_{p(p)m(t)} + \varphi_{sy(t)} + \upsilon_{sy(t)} + cb(SPEI\!-3, l) + cb(Pre, l)$ | 10841.02 | 1.360 |

Note: The baseline model comprised an intercept, α, province-specific monthly random effects, $\beta_{p(p)m(t)}$, year-specific spatially unstructured, $\varphi_{sy(t)}$, and structured, $\upsilon_{sy(t)}$, random effects at the city level. The final model additionally added the cross-basis functions of SPEI-3, $cb(SPEI\!-3, l)$, and $T_{min}$, $cb(T_{min}, l)$, with lags, *l*, from zero to six months. DIC, deviance information criterion; CV, cross-validated; SPEI, standardized precipitation evapotranspiration index; cb, cross-basis. $T_{mean}$, monthly mean temperature; $T_{max}$, monthly maximum temperature; $T_{min}$, monthly minimum temperature; Pre, monthly precipitation.

© 2023 Li C et al. *JAMA Network Open.*

**eFigure 1. Spatial distribution of annual mean value of city development indicators for the 54 cities of the four provinces in China, 2013-2019**

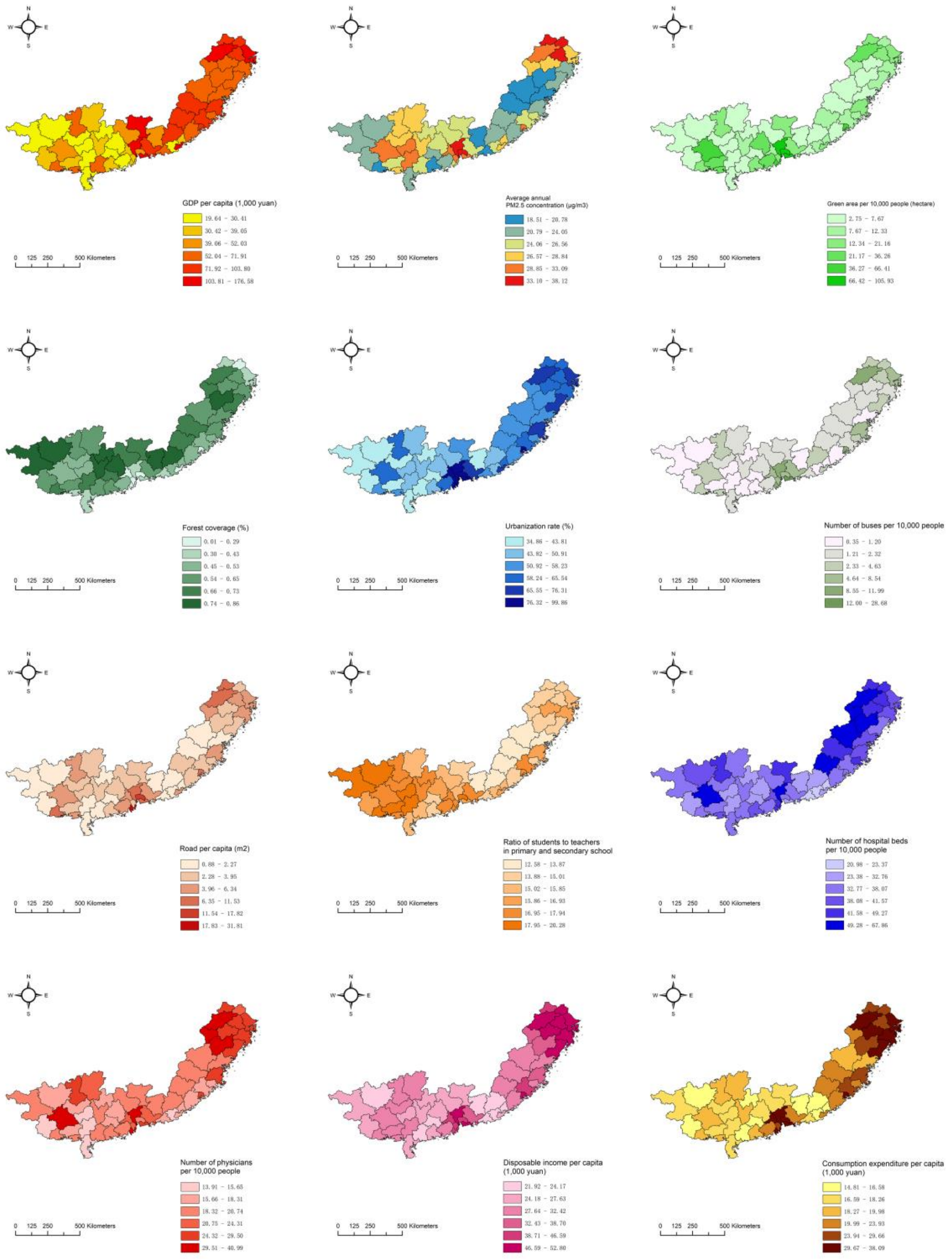


© 2023 Li C et al. *JAMA Network Open.*

## eFigure 2. The flowchart of model construction procedure

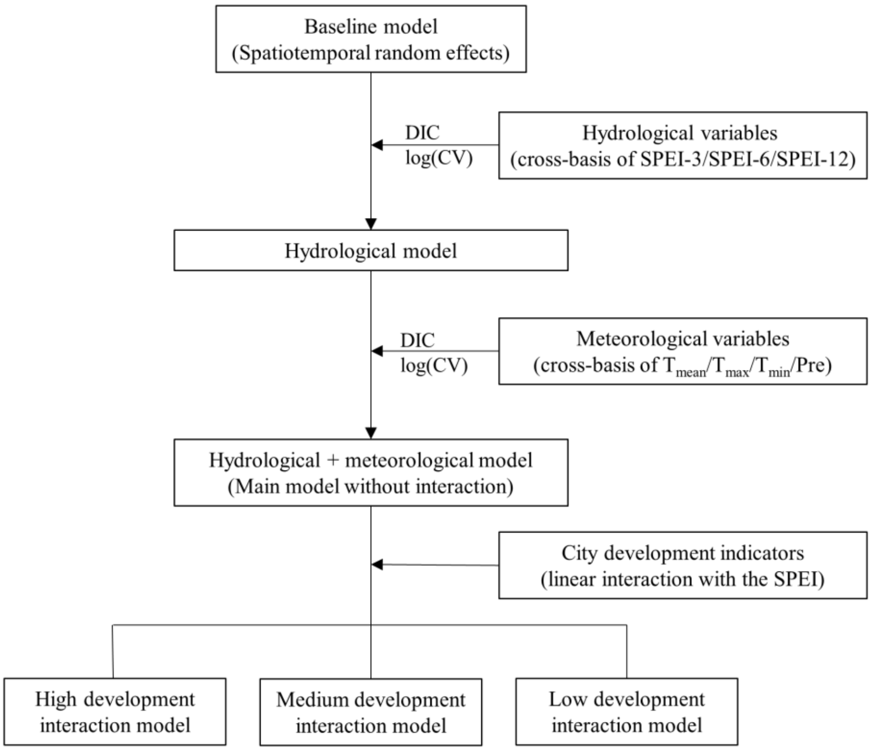


© 2023 Li C et al. *JAMA Network Open.*

**eFigure 3. The annual dengue incidence in the four provinces in China, 2013-2019**

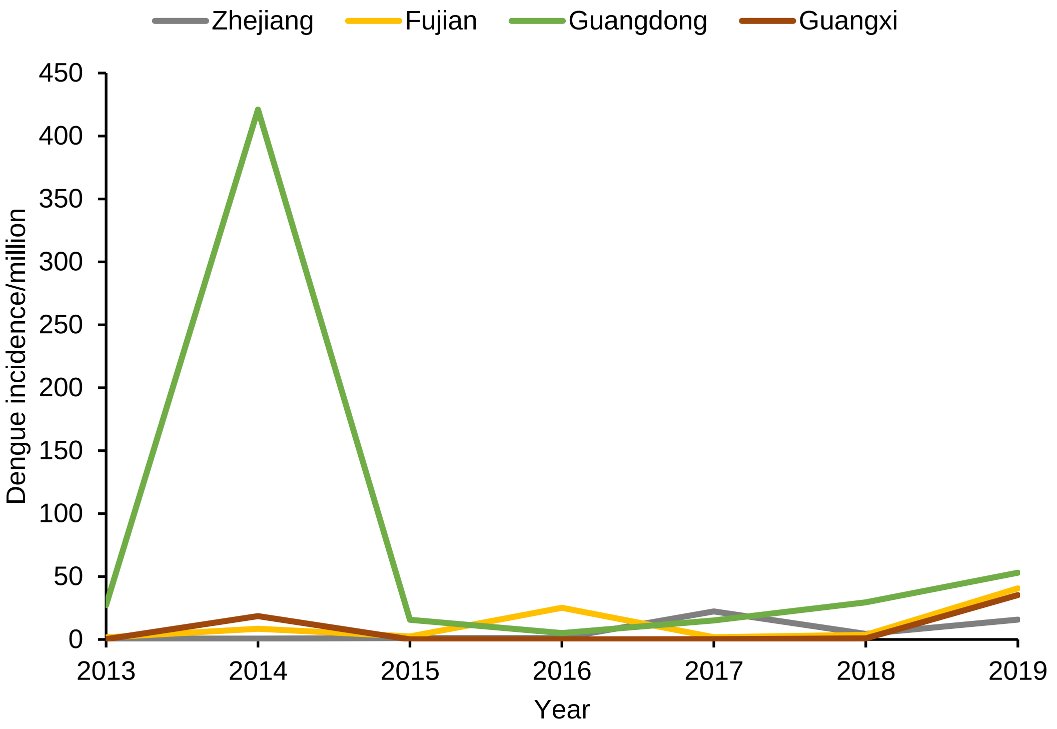


© 2023 Li C et al. *JAMA Network Open.*

**eFigure 4. Monthly dengue incidence rates (per 10 000 000 population) at provincial level, 2013-2019**

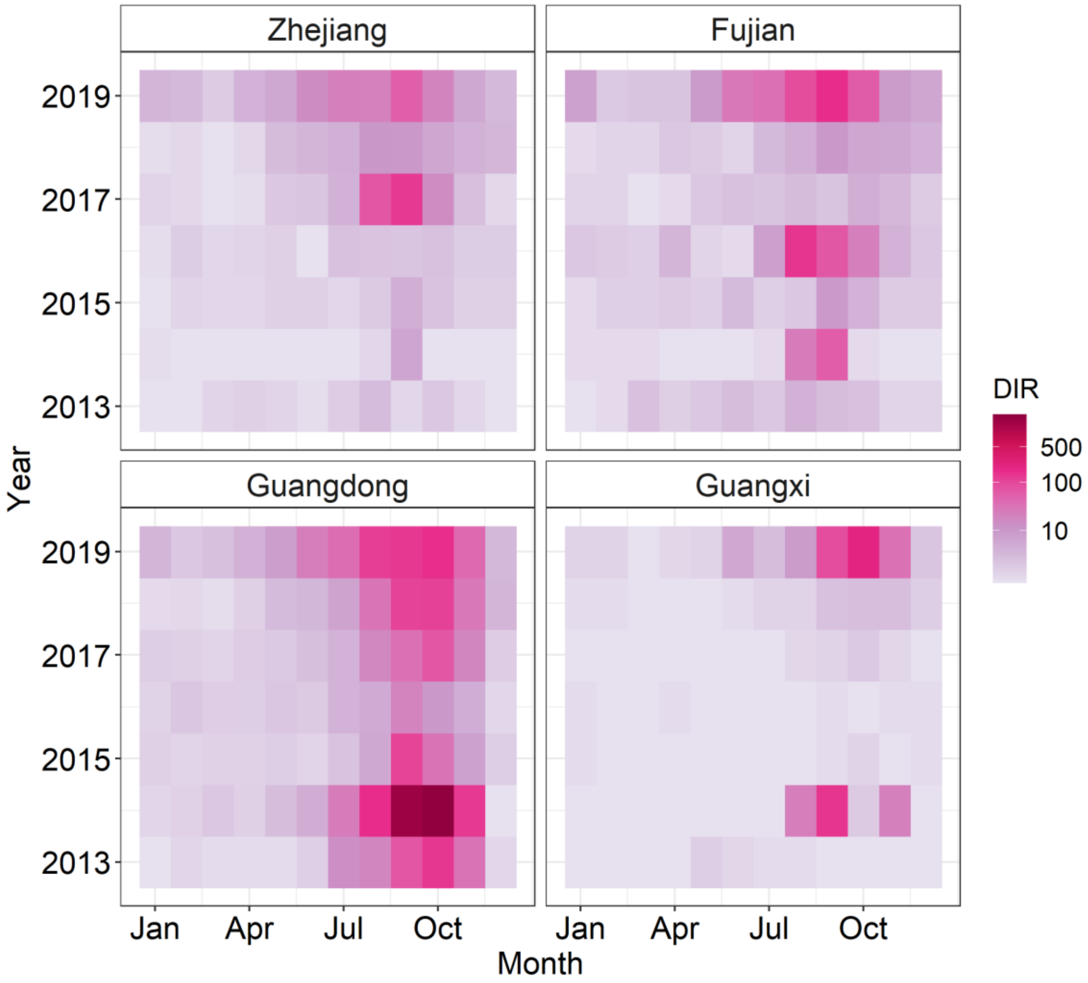


Note: DIR, dengue incidence rate.

© 2023 Li C et al. *JAMA Network Open.*

## eFigure 5. Monthly SPEI-3, -6, and -12 at provincial level, 2013-2019

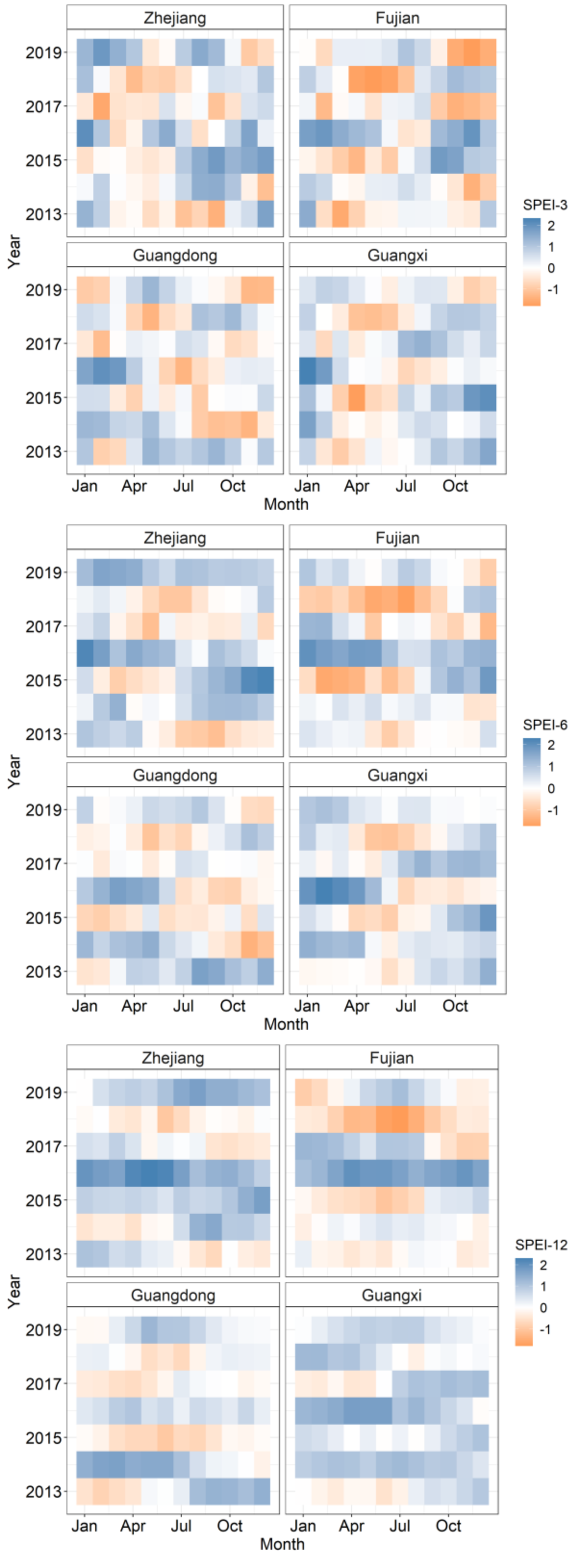


Note: SPEI, standardized precipitation evapotranspiration index.

© 2023 Li C et al. *JAMA Network Open.*

## eFigure 6. Monthly $T_{mean}$, $T_{max}$, $T_{min}$ (˚C), and precipitation (mm) at provincial level, 2013-2019

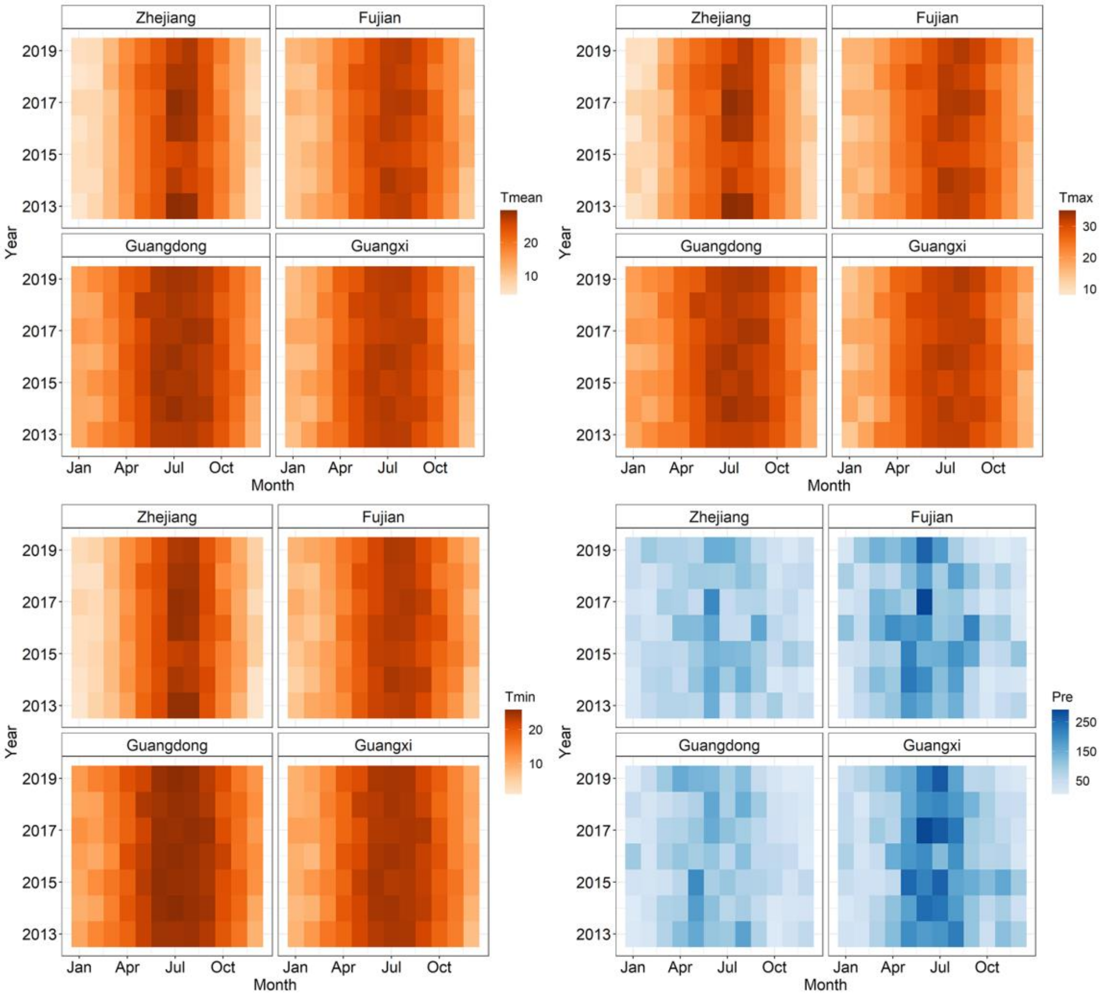


Note: $T_{mean}$, mean temperature; $T_{max}$, maximum temperature; $T_{min}$, minimum temperature; Pre, precipitation.

© 2023 Li C et al. *JAMA Network Open.*

## eFigure 7. Relative risk of dengue with $T_{min}$ exposures and lags in the four provinces in China

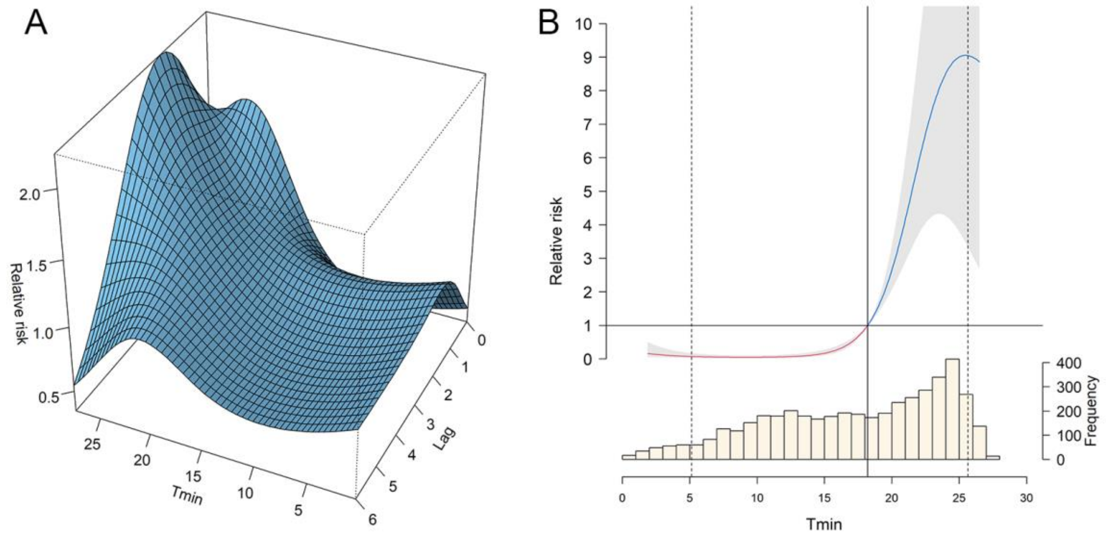


(A) Three-dimensional plot of the exposure-lag-response association between $T_{min}$ and dengue incidence relative to median $T_{min}$ (18.2 ˚C); (B) Overall cumulative exposure-response association between $T_{min}$ and dengue incidence vs median $T_{min}$ within a 6-month lag.
Note: $T_{min}$, monthly minimum temperature.

© 2023 Li C et al. *JAMA Network Open.*

## eFigure 8. Sensitivity results of cumulative relative risk of SPEI-3 on dengue incidence

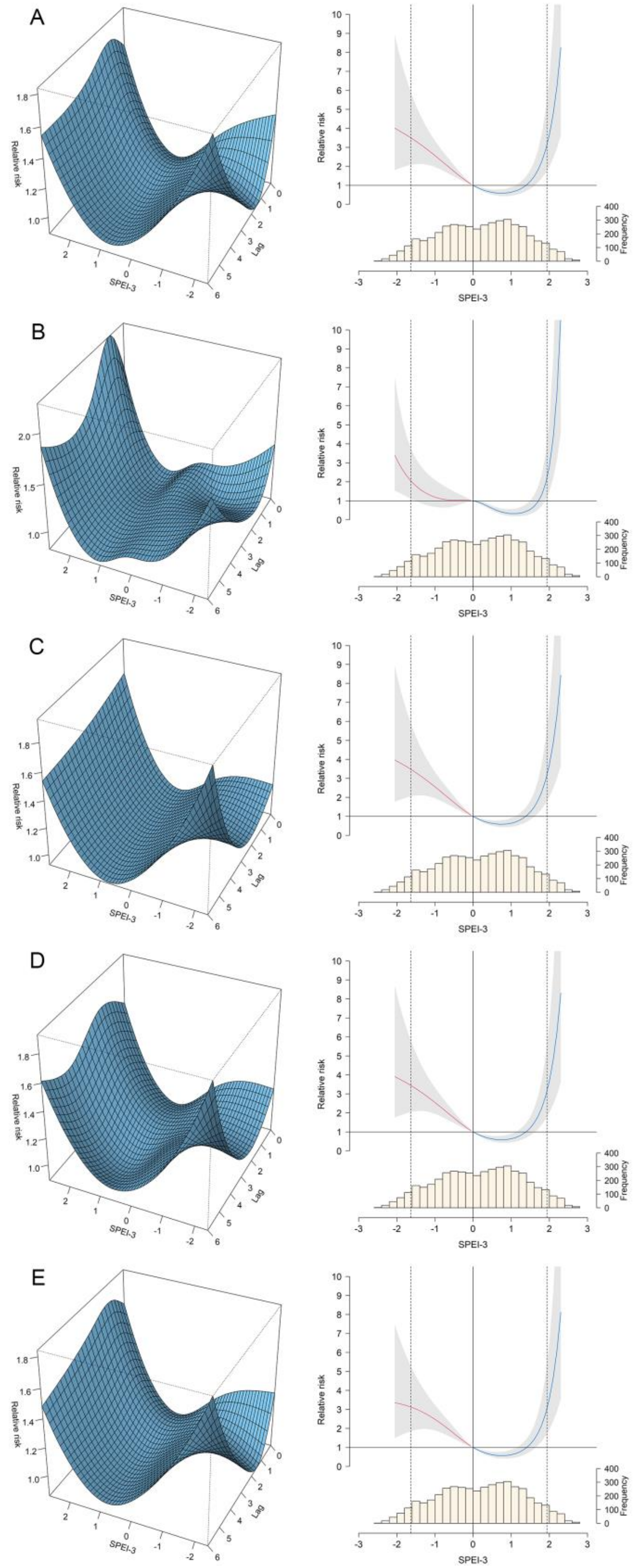

(A) main model; (B) changing the knots number of exposure dimension in the cross basis of SPEI-3 to three; (C) changing the knots number of lag dimension in the cross basis of SPEI-3 to one; (D) changing the knots of lag dimension in the cross basis of SPEI-3 to equidistant distribution; (E) changing $T_{min}$ to $T_{mean}$ variable.

© 2023 Li C et al. *JAMA Network Open.*

**eFigure 9. Lag-response associations for extreme wet and extreme dry conditions under high- and low-economic and public services development scenario**

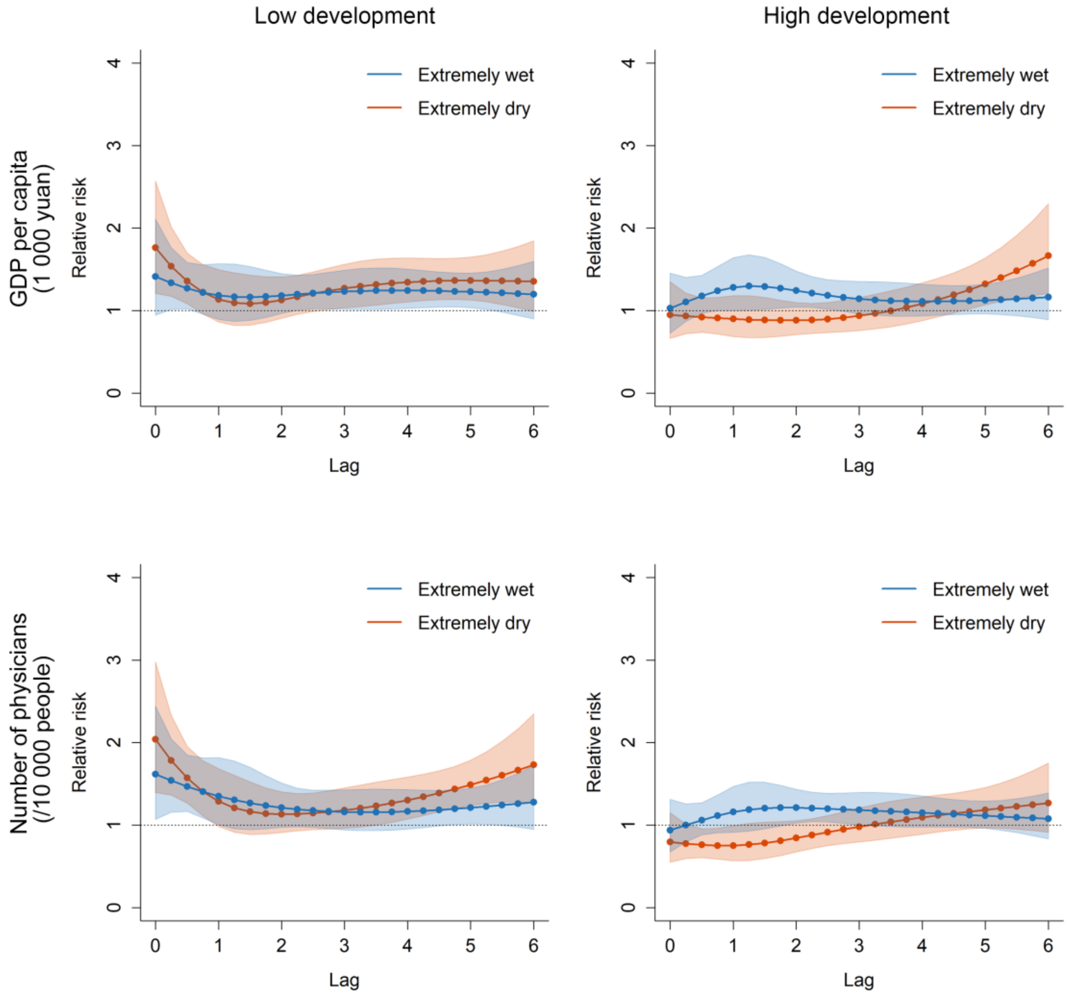


Note: Extreme wet condition had a Standardized Precipitation Evapotranspiration Index (SPEI)-3 of 2, and extreme dry condition had an SPEI-3 of −2. To convert yuan to US dollars, multiply by 0.14.

© 2023 Li C et al. *JAMA Network Open.*

**eFigure 10. Contour plots of the exposure-lag-response associations between the SPEI-3 and dengue incidence overall and under high-, middle-, and low-development scenario**

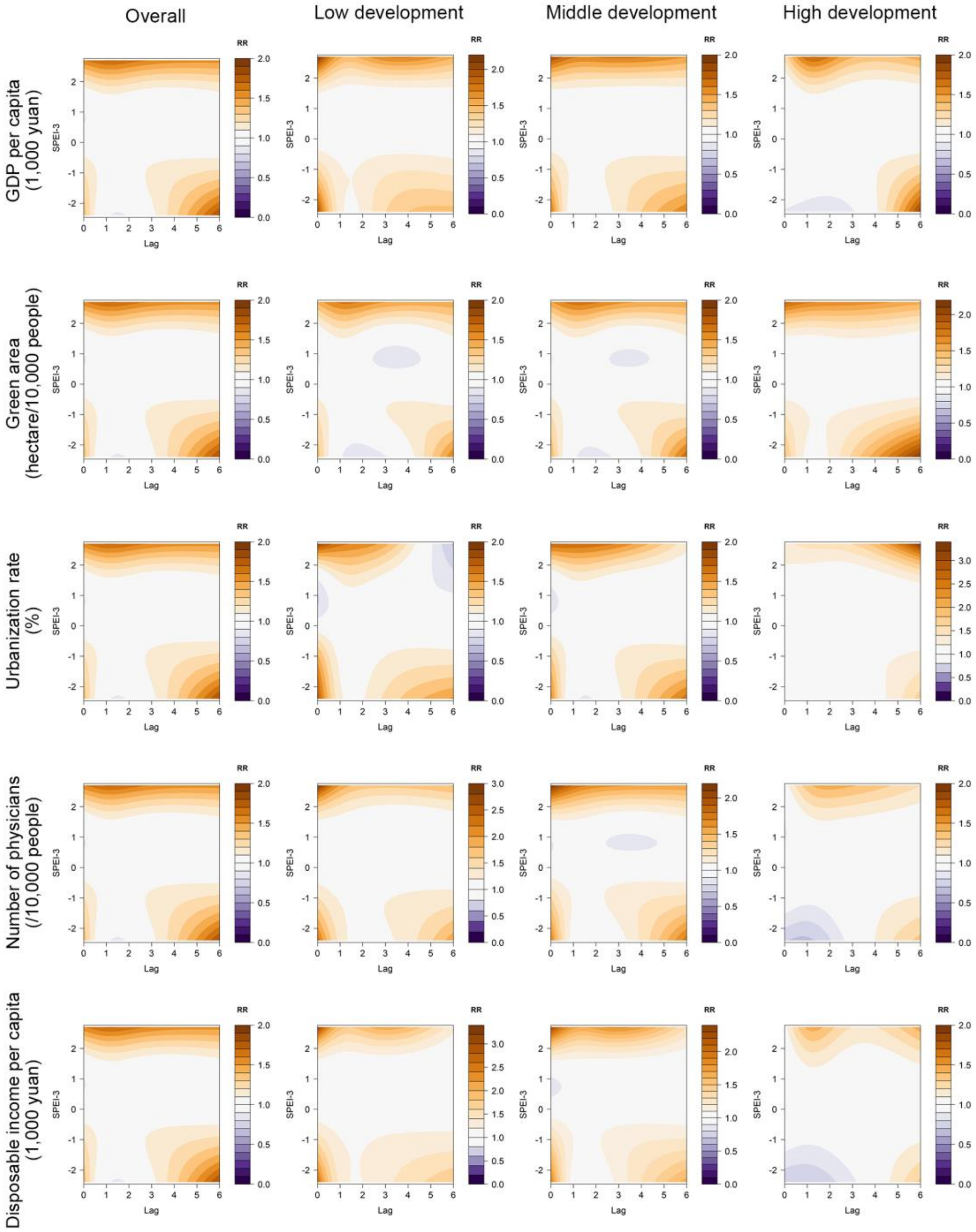


© 2023 Li C et al. *JAMA Network Open.*

## eReferences


1. Palmer WC. Meteorological drought. Research Paper No. 45, US Weather Bureau, Washington, DC. 1965.
2. Vicente-Serrano SM, Beguería S, López-Moreno J. A multiscalar drought index sensitive to global warming: the standardized precipitation evapotranspiration index. *J Clim.* 2010;23(7):1696-1718. doi:10.1175/2009JCLI2909.1
3. Mckee TB, Doesken NJ, Kleist J. The relationship of drought frequency and duration to time scales. Eighth Conference on Applied Climatology; 1993.
4. Ault TR. On the essentials of drought in a changing climate. *Science.* 2020;368(6488):256-260. doi:10.1126/science.aaz5492
5. Adams HD, Guardiola-Claramonte M, Barron-Gafford GA, et al. Temperature sensitivity of drought-induced tree mortality portends increased regional die-off under global-change-type drought. *Proc Natl Acad Sci U S A.* 2009;106(17):7063-7066. doi:10.1073/pnas.0901438106
6. Barriopedro D, Fischer EM, Luterbacher J, Trigo RM, García-Herrera R. The hot summer of 2010: redrawing the temperature record map of Europe. *Science.* 2011;332(6026):220-224. doi:10.1126/science.1201224
7. Vicente-Serrano SM. Differences in spatial patterns of drought on different time scales: an analysis of the Iberian Peninsula. *Water Resour Manag.* 2006;20(1):37-60.
8. Alam I, Otani S, Nagata A, et al. Short- and long-term effects of drought on selected causes of mortality in northern Bangladesh. *Int J Environ Res Public Health.* 2022;19(6). doi:10.3390/ijerph19063425
9. Thornthwaite CW. An approach toward a rational classification of climate. *Geogr Rev.* 1948;38:55-94.
10. Ahmad MI, Sinclair CD, Werritty A. Log-logistic flood frequency analysis. *J Hydrol.* 1988;98(3-4):205-224.
11. Hosking J. L-Moments: Analysis and estimation of distributions using linear combinations of order statistics. *J R Statist Soc B.* 1990;52(1):105-124.


© 2023 Li C et al. *JAMA Network Open.*
