# Supplementary material for: Association Between Hydrological Conditions and Dengue Fever Incidence in Coastal Southeastern China From 2013 to 2019
Source: JAMA Netw Open. 2023 Jan 4;6(1):e2249440. doi: 10.1001/jamanetworkopen.2022.49440 (PMC9857674; doi:10.1001/jamanetworkopen.2022.49440)
Supplement: Supplement 2. — Data Sharing Statement [file jamanetwopen-e2249440-s002.pdf]

Data Sharing Statement

Li. Association Between Hydrological Conditions and Dengue Fever Incidence in Coastal Southeastern China From 2013 to 2019. *JAMA Netw Open*. Published January 04, 2023. doi:10.1001/jamanetworkopen.2022.49440

Data
Data available: No

Additional Information
Explanation for why data not available: The dengue data underlying the results presented in the study cannot be shared publicly because of the limitation of data availability in the data management rule of Chinese Center for Disease Control and Prevention. Access to these data may be requested through the Chinese Center for Disease Control and Prevention for researchers who meet the criteria for access to confidential data.
